# Supplementary material for: The Effects of Commonly Consumed Dietary Fibres on the Gut Microbiome and Its Fibre Fermentative Capacity in Adults with Inflammatory Bowel Disease in Remission
Source: Nutrients. 2022 Mar 2;14(5):1053. doi: 10.3390/nu14051053 (PMC8912623; doi:10.3390/nu14051053)

Healthy Controls: Non-Substrate Control

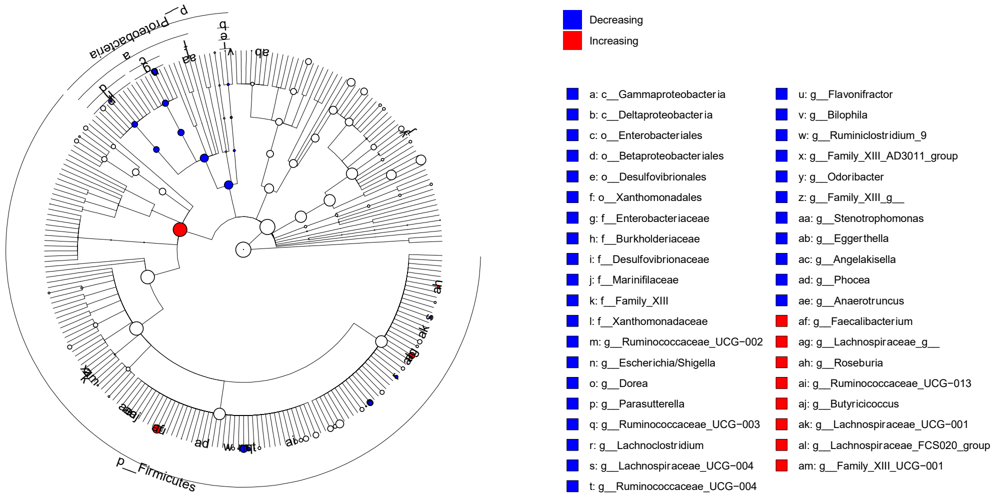

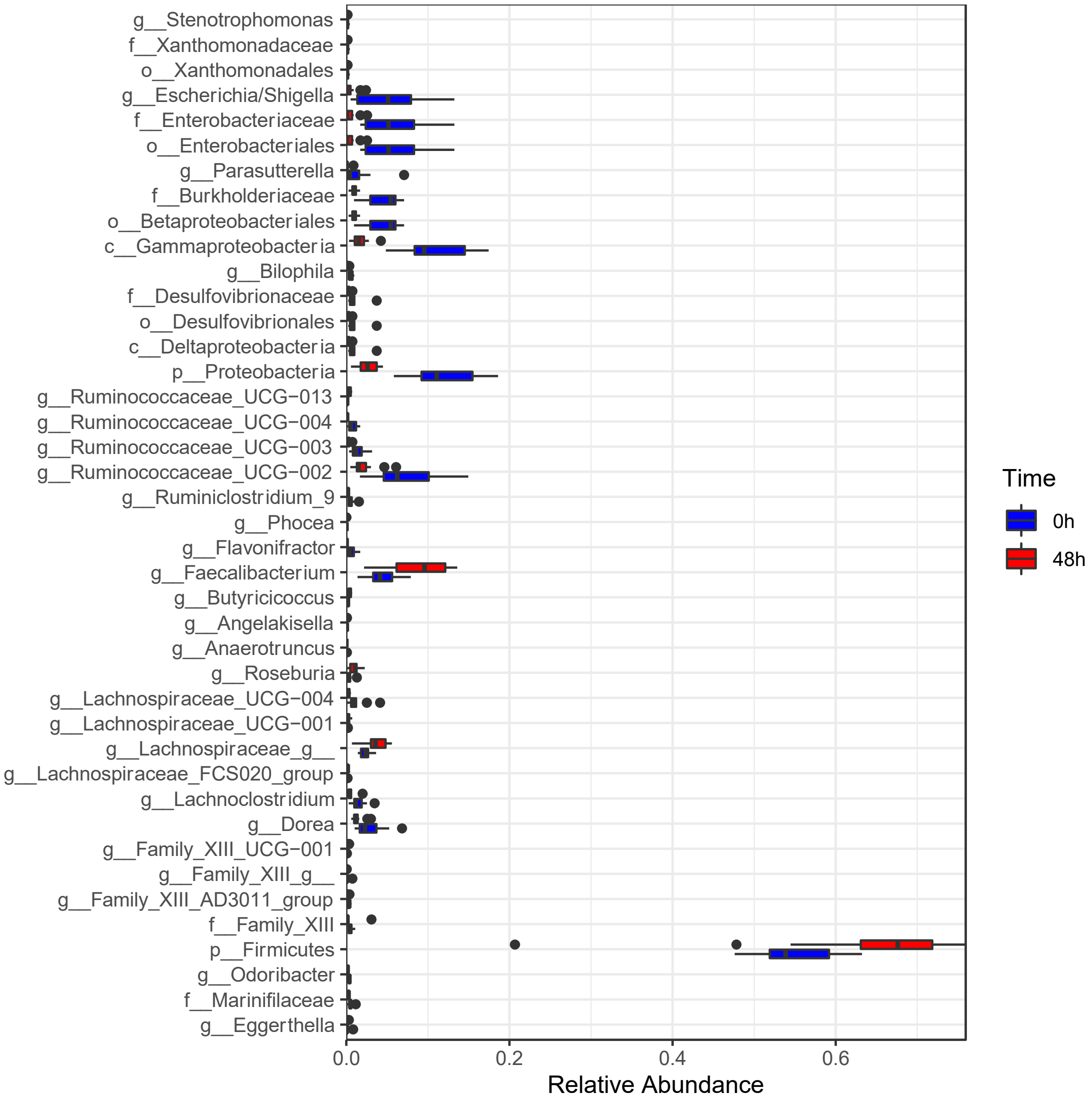
g__Stenotrophomonas
f__Xanthomonadaceae
o__Xanthomonadales
g__Escherichia/Shigella
f__Enterobacteriaceae
o__Enterobacteriales
g__Parasutterella
f__Burkholderiaceae
o__Betaproteobacteriales
c__Gammaproteobacteria
g__Bilophila
f__Desulfovibrionaceae
o__Desulfovibrionales
c__Deltaproteobacteria
p__Proteobacteria
g__Ruminococcaceae_UCG−013
g__Ruminococcaceae_UCG−004
g__Ruminococcaceae_UCG−003
g__Ruminococcaceae_UCG−002
g__Ruminiclostridium_9
g__Phocea
g__Flavonifractor
g__Faecalibacterium
g__Butyricicoccus
g__Angelakisella
g__Anaerotruncus
g__Roseburia
g__Lachnospiraceae_UCG−004
g__Lachnospiraceae_UCG−001
g__Lachnospiraceae_g__
g__Lachnospiraceae_FCS020_group
g__Lachnoclostridium
g__Dorea
g__Family_XIII_UCG−001
g__Family_XIII_g__
g__Family_XIII_AD3011_group
f__Family_XIII
p__Firmicutes
g__Odoribacter
f__Marinifilaceae
g__Eggerthella
0.0
0.2
0.4
0.6
Relative Abundance
Time
0h
48h

Healthy Controls: Hi Maize

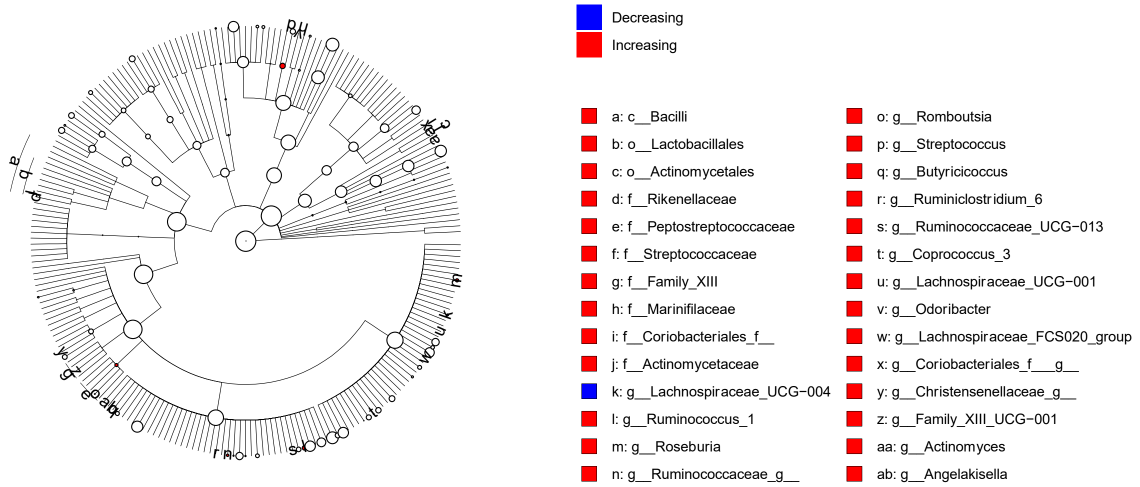

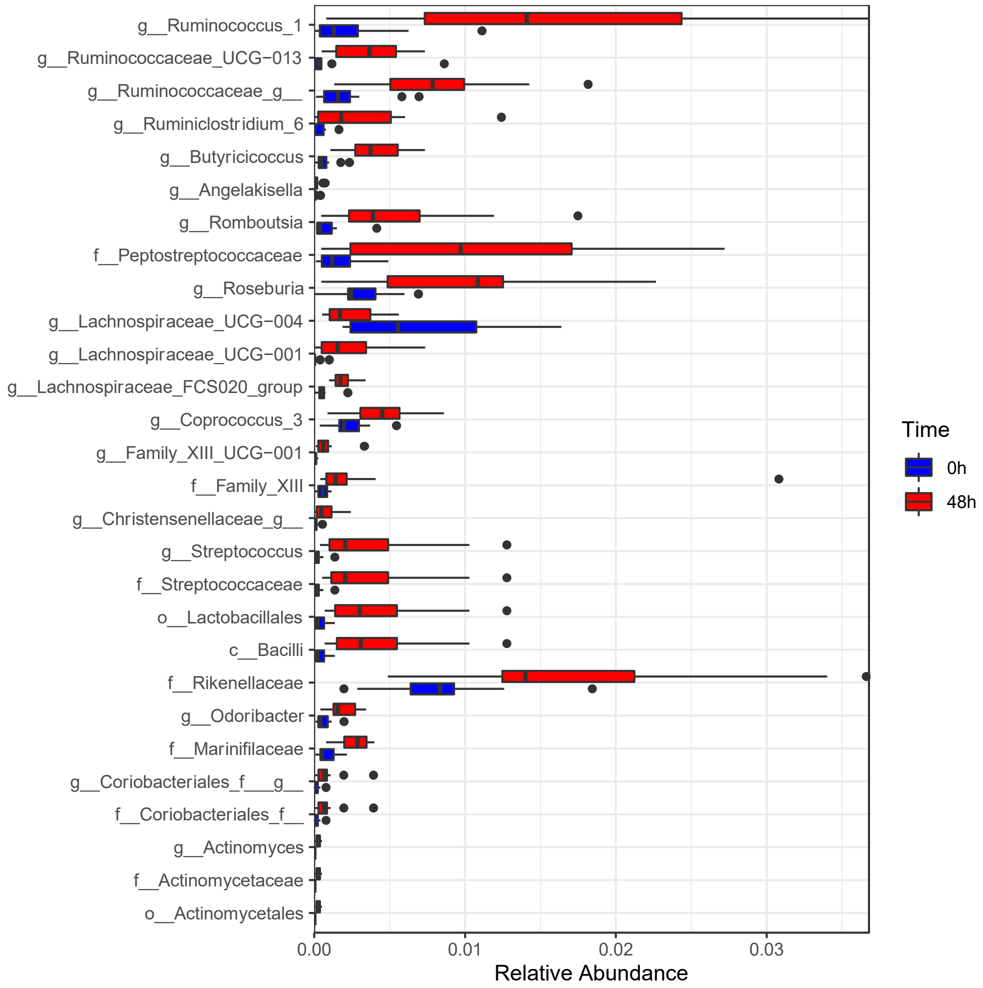

Healthy Controls: Apple Pectin

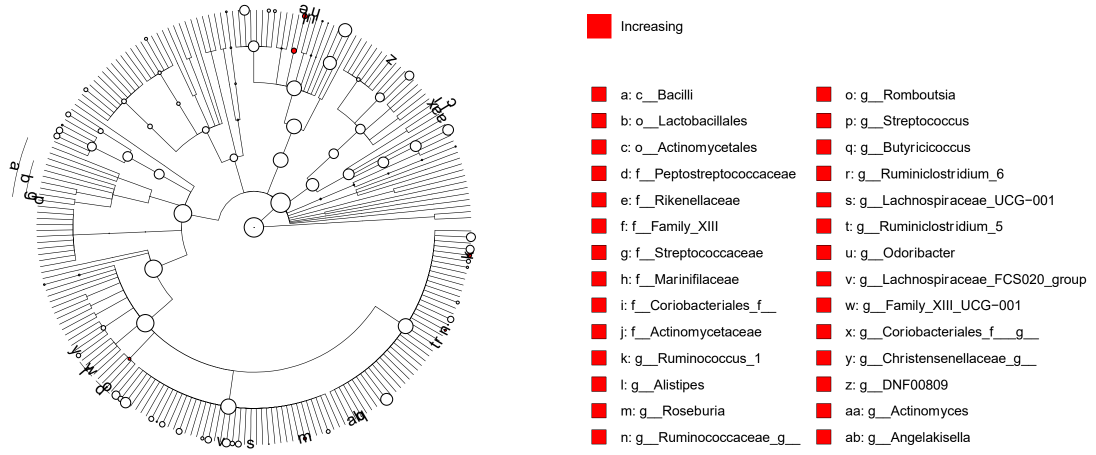

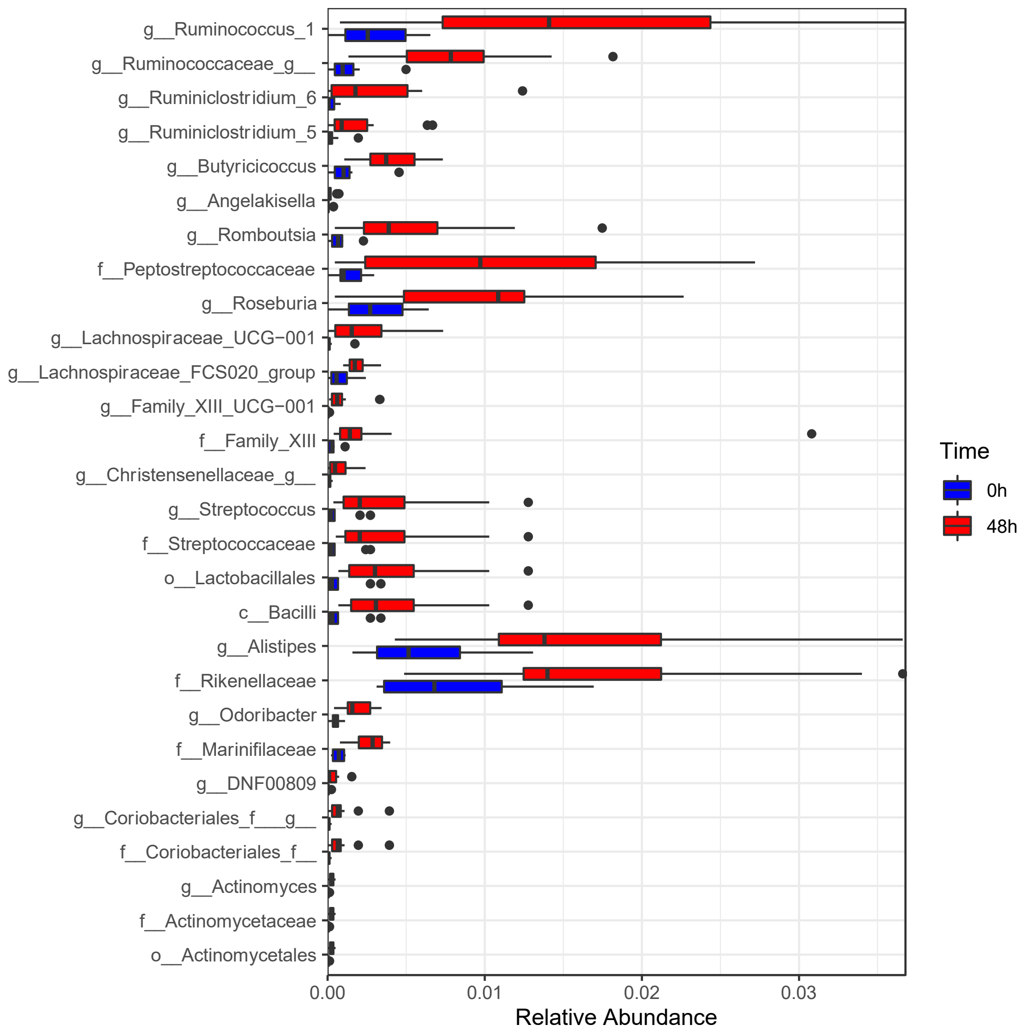

Healthy Controls: Raftilose

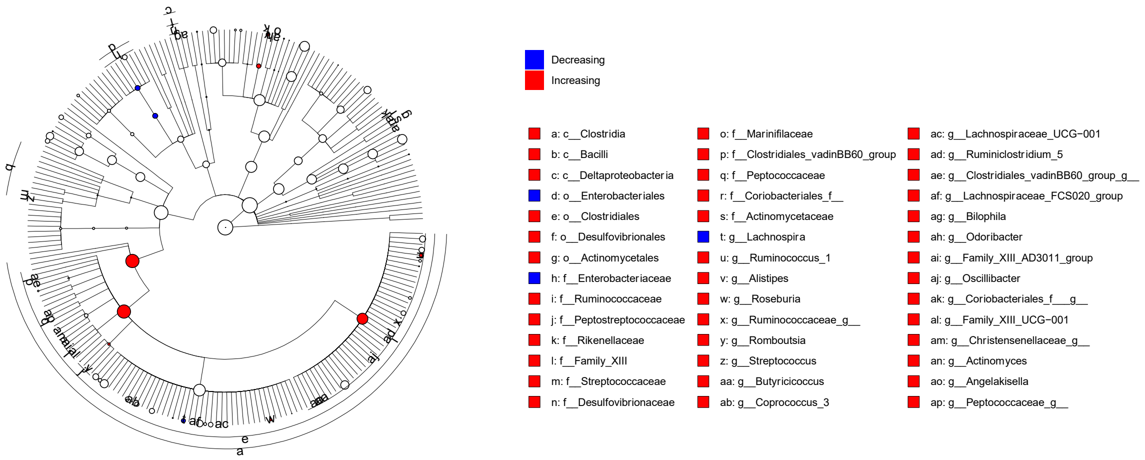

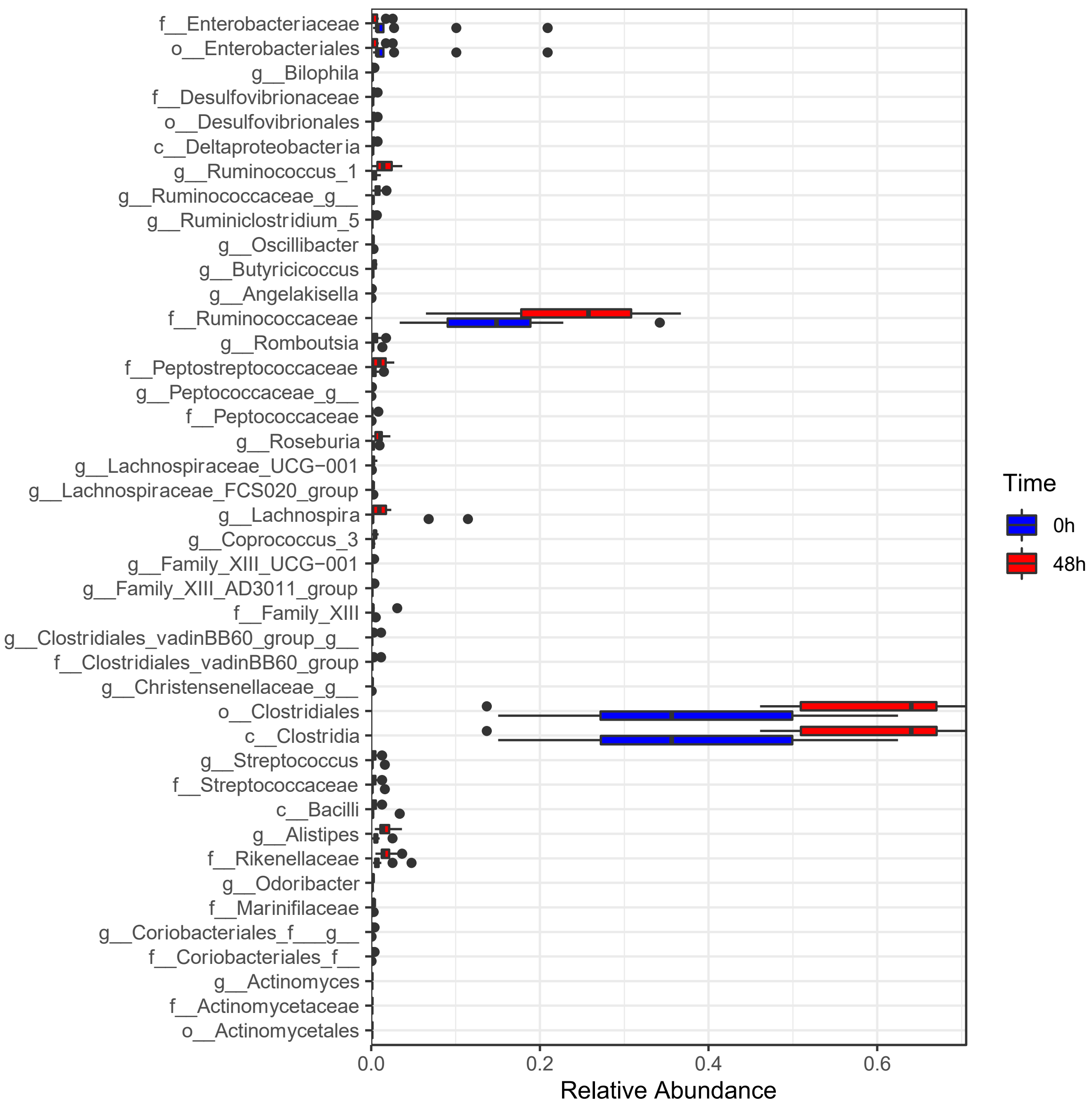

Healthy Controls: Wheat Bran

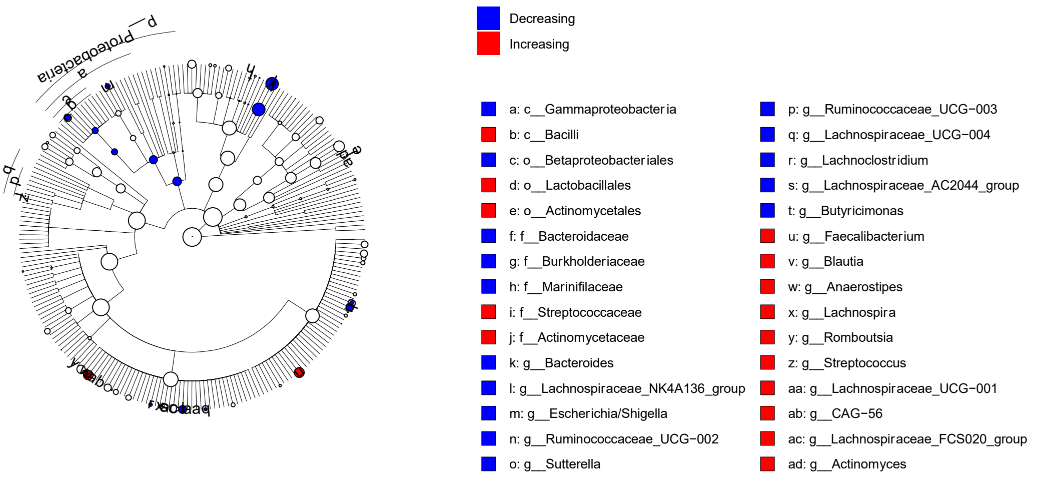

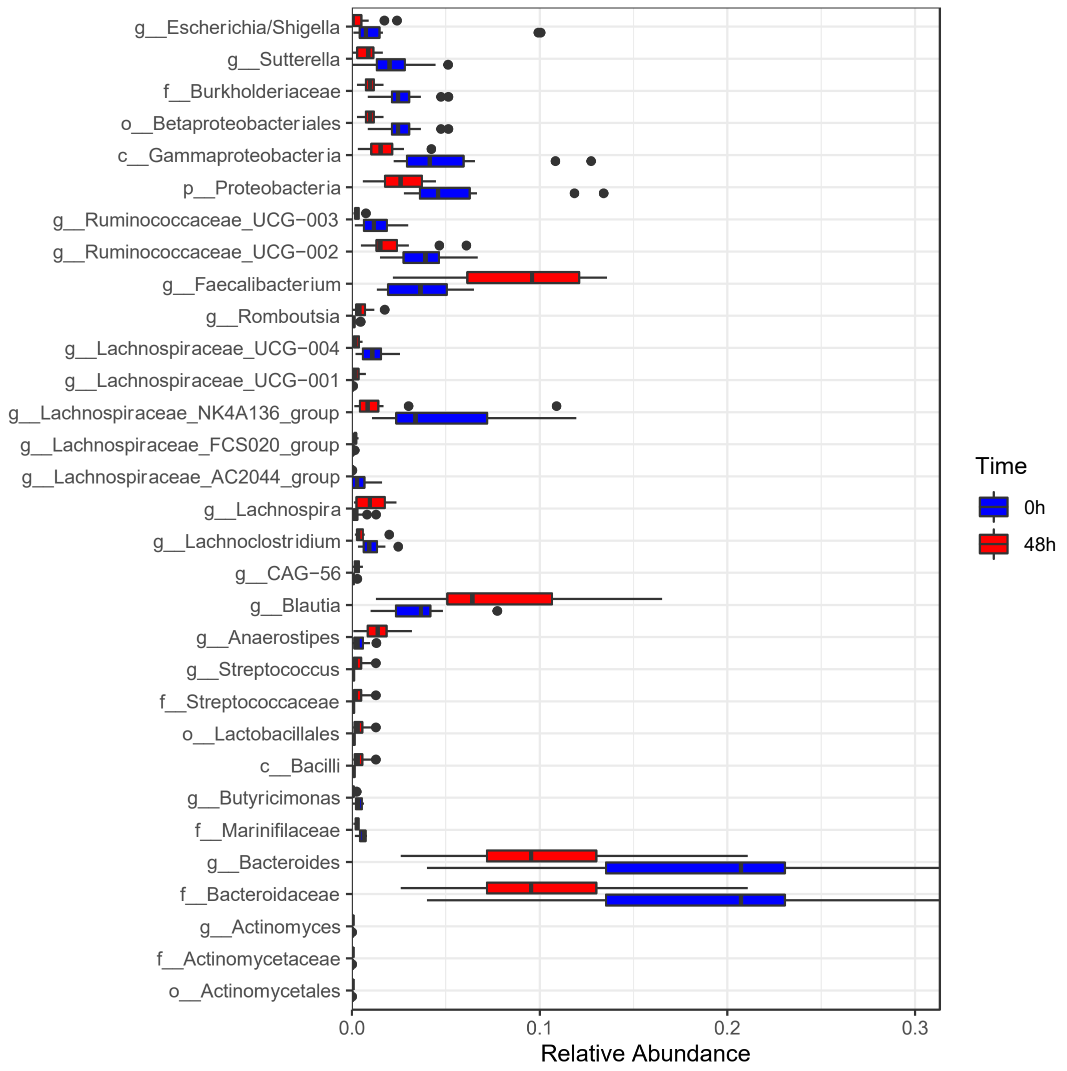

Healthy Controls: Mixed Fibre

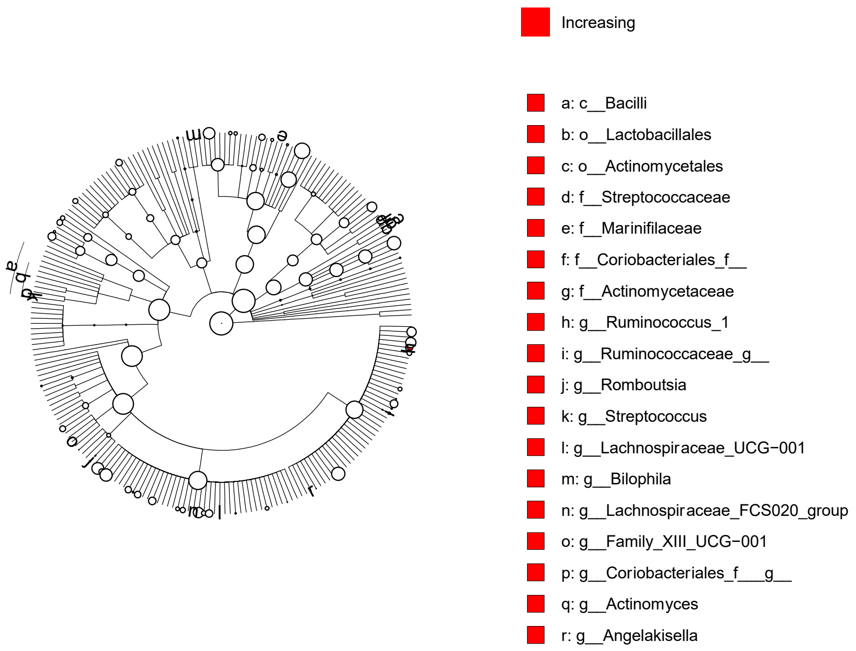

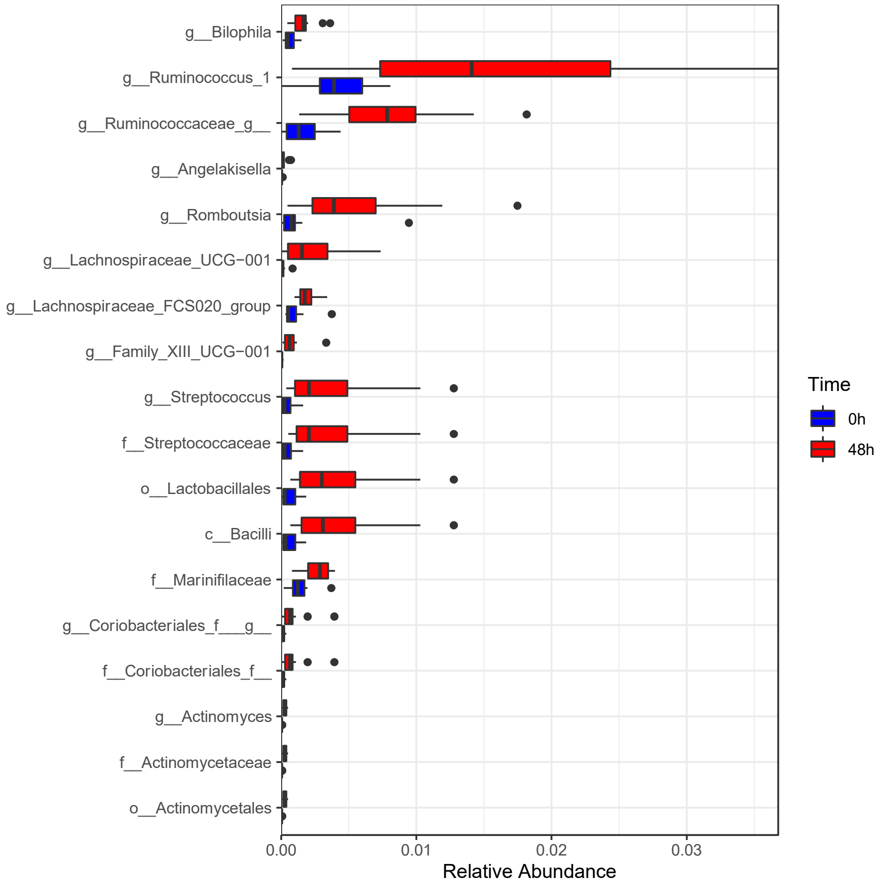

Ulcerative Colitis: Non-substrate control

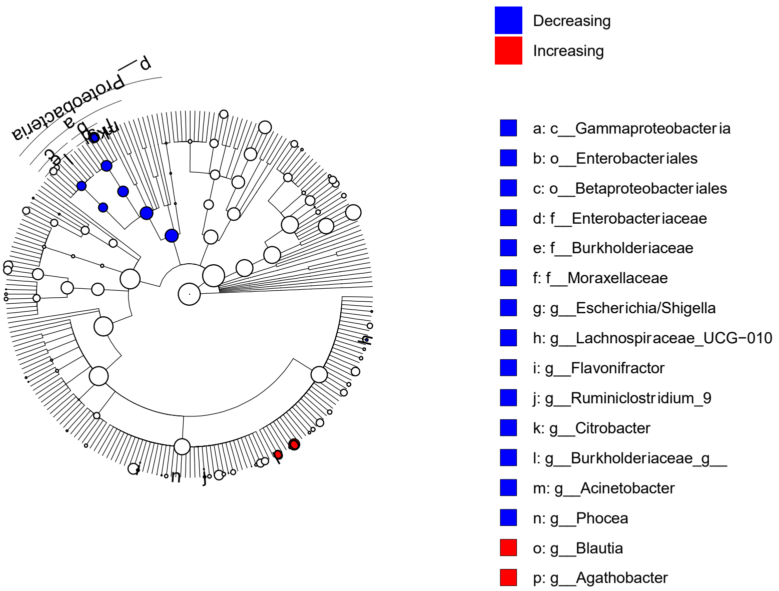

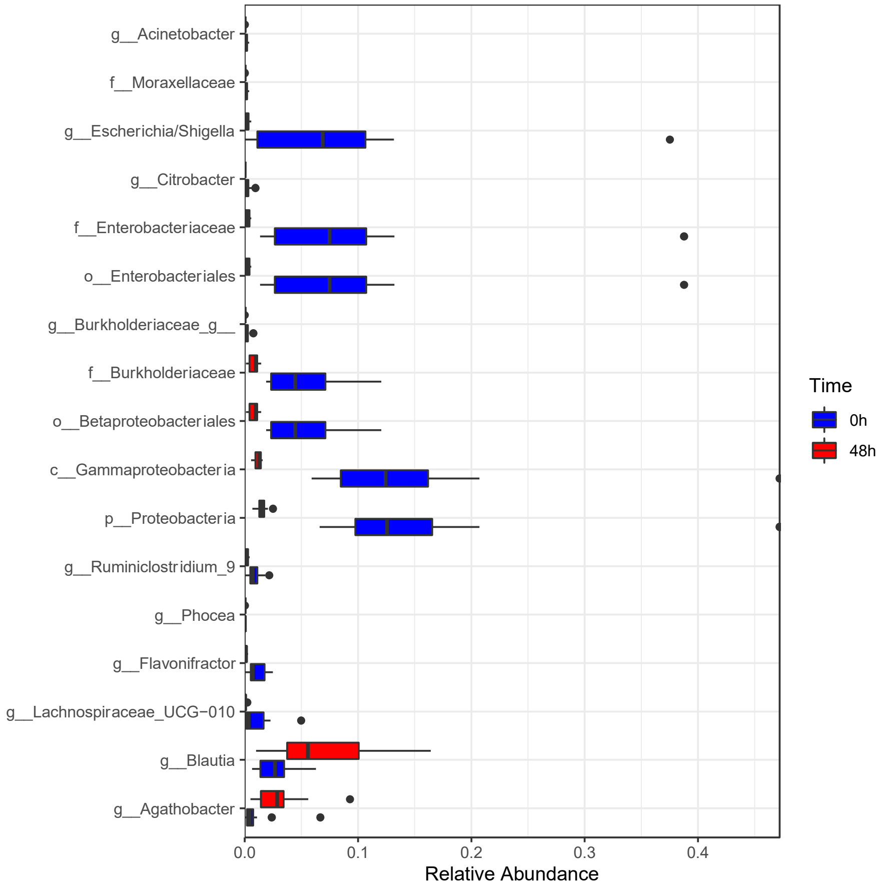

Ulcerative Colitis: Raftilose

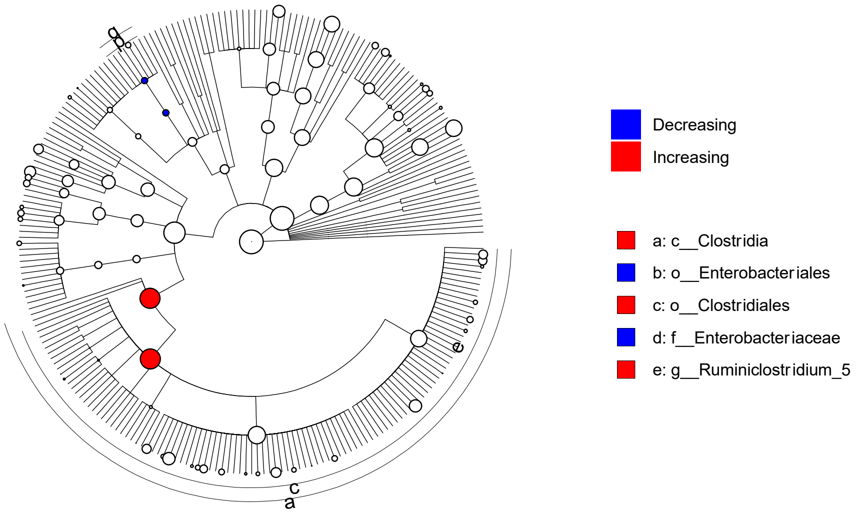

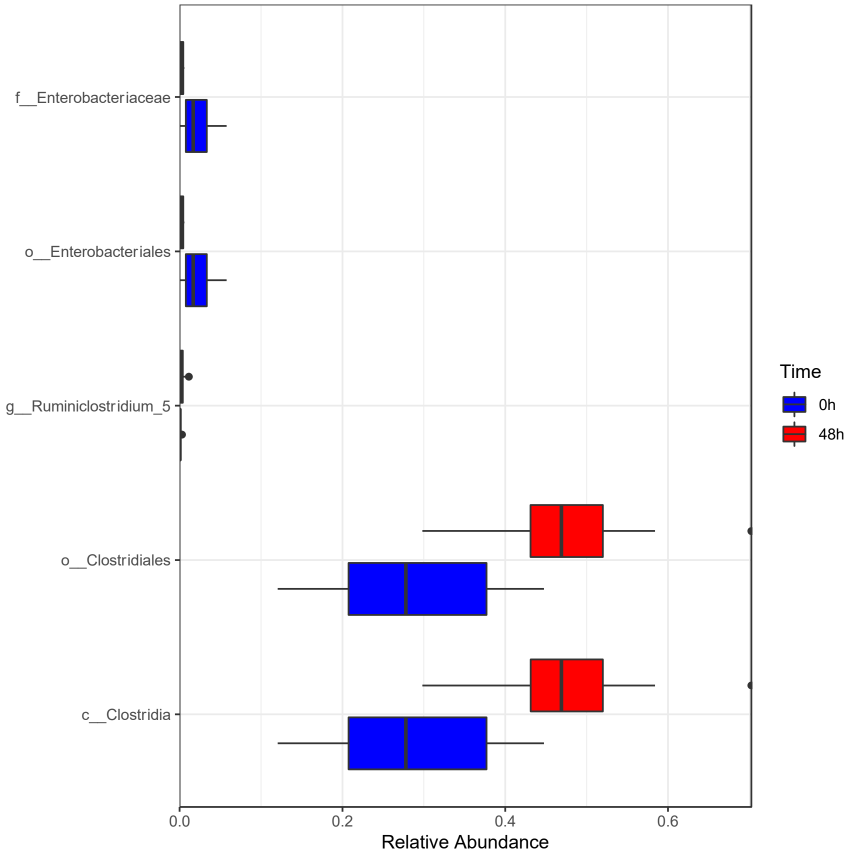

Ulcerative Colitis: Wheat Bran

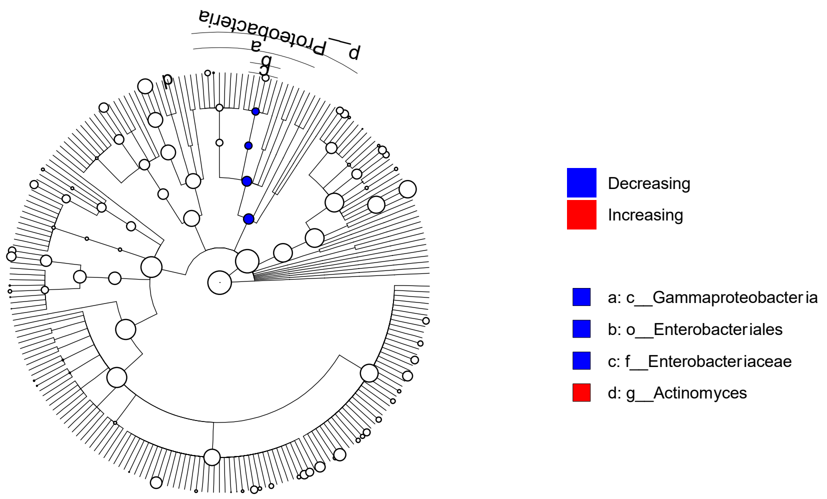

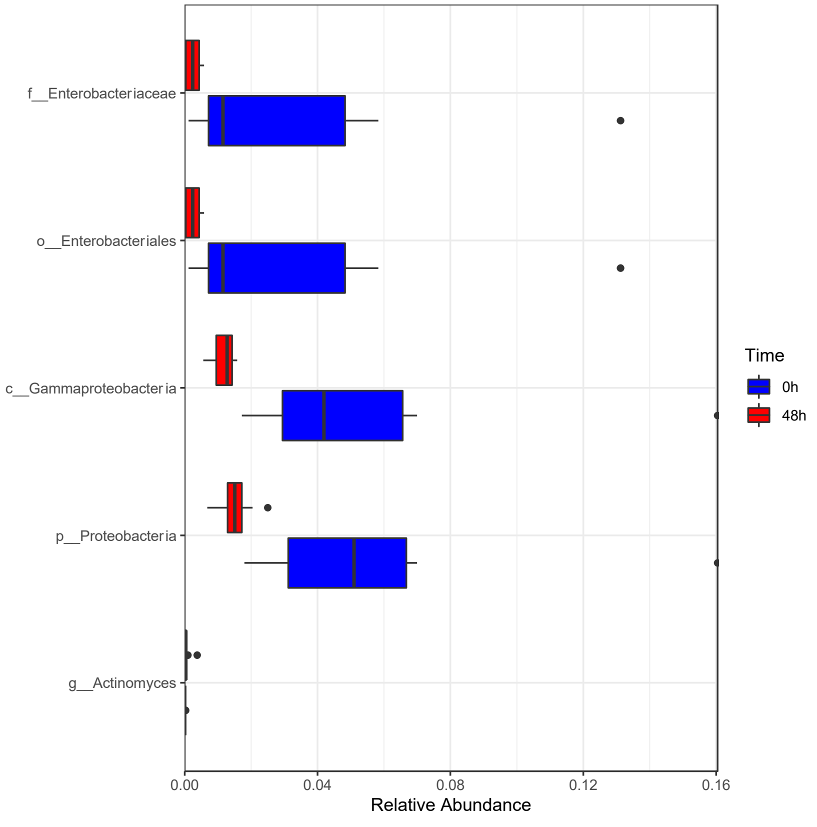

Crohn’s Disease: Mixed Fibre

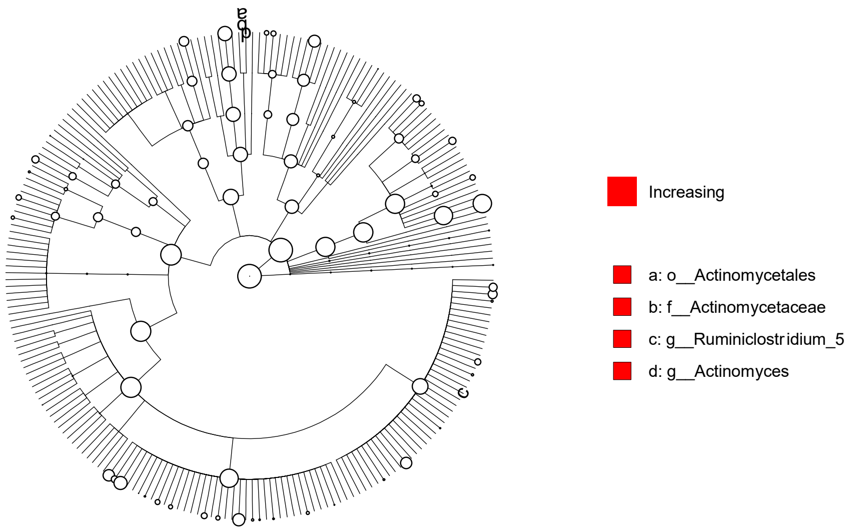

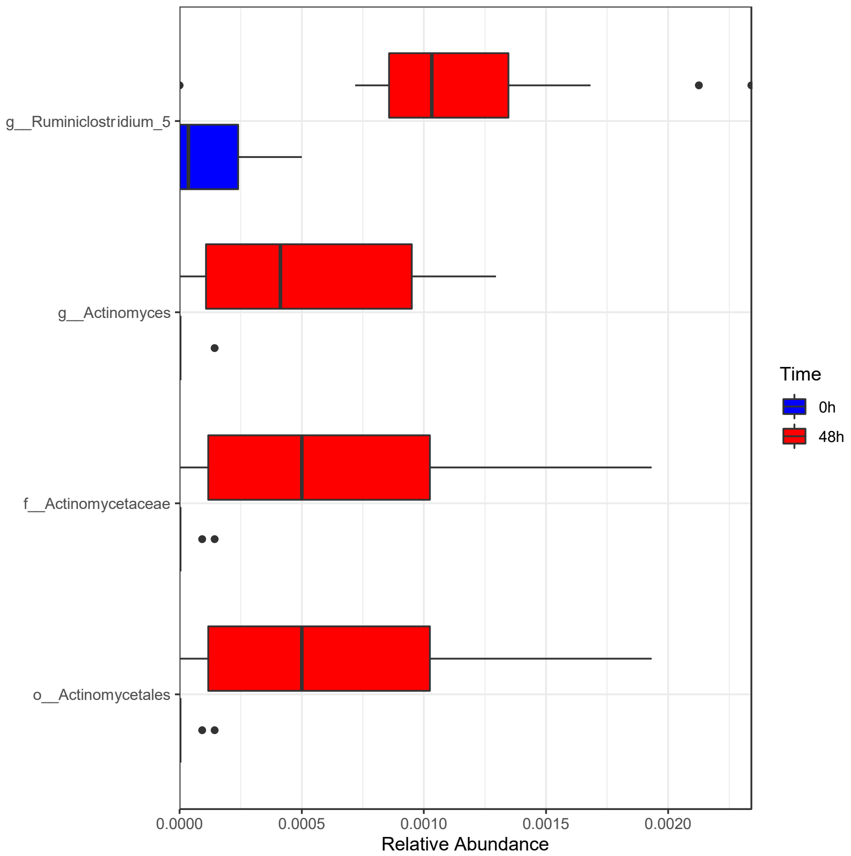

Supplement: Supplementary file 1 [file nutrients-14-01053-s001.zip › Figure S4.pdf]
