# Supplementary material for: The Effects of Commonly Consumed Dietary Fibres on the Gut Microbiome and Its Fibre Fermentative Capacity in Adults with Inflammatory Bowel Disease in Remission
Source: Nutrients. 2022 Mar 2;14(5):1053. doi: 10.3390/nu14051053 (PMC8912623; doi:10.3390/nu14051053)
Supplement: Supplementary file 1 [file nutrients-14-01053-s001.zip › Table S1.pdf]

Table S1: Concentration of faecal short and branched chain fatty acids in adults with Crohn's disease and ulcerative colitis in remission and compared with healthy controls

	**Healthy Controls**	**Crohn's Disease**	**Ulcerative Colitis**
N	8	8	8
Faecal water (%)	64 (61 : 70)	71 (65 : 79)	73 (68 : 78)
Acetate	83 (67 : 101)	82 (59 : 112)	73 (63 : 114)
Propionate	16.3 (14.2 : 23.3)	16.8 (13.1 : 22)	12.8 (10.8 : 24.9)
Butyrate	15.5 (12.8 : 16.8)	10.3 (7.6 : 24.7)	15.2 (7.5 : 23)
Valerate	1.9 (1.7 : 3.0)	2.3 (1.5 : 2.7)	1.8 (1.4 : 2.8)
Hexanoate	0.8 (0.3 : 1.4)	1.0 (0.6 : 1.3)	0.2 (0.1 : 0.4)*
Heptanoate	0.11 (0.04 : 0.23)	0.16 (0.07 : 0.22)	0.05 (0.02 : 0.07)
Octanoate	0.11 (0.04 : 0.51)	0.06 (0.04 : 0.1)	0.18 (0.04 : 0.4)
iso-butyrate	1.6 (1.5 : 2.8)	1.8 (1.5 : 2.4)	1.7 (1.2 : 2.1)
iso-valerate	2.2 (1.8 : 4.3)	2.6 (1.9 : 3.1)	2.2 (2.0 : 2.6)
iso-hexanoate	0.07 (0.06 : 0.09)	0.07 (0.06 : 0.09)	0.08 (0.04 : 0.11)
Total SCFA	126 (102 : 147)	106 (89 : 167)	117 (93 : 158)

Data (µmol/g of faeces) are presented with median and inter-quartile ranges
