# Supplementary figures and images for: The Effects of Commonly Consumed Dietary Fibres on the Gut Microbiome and Its Fibre Fermentative Capacity in Adults with Inflammatory Bowel Disease in Remission
Source: Nutrients. 2022 Mar 2;14(5):1053. doi: 10.3390/nu14051053 (PMC8912623; doi:10.3390/nu14051053)

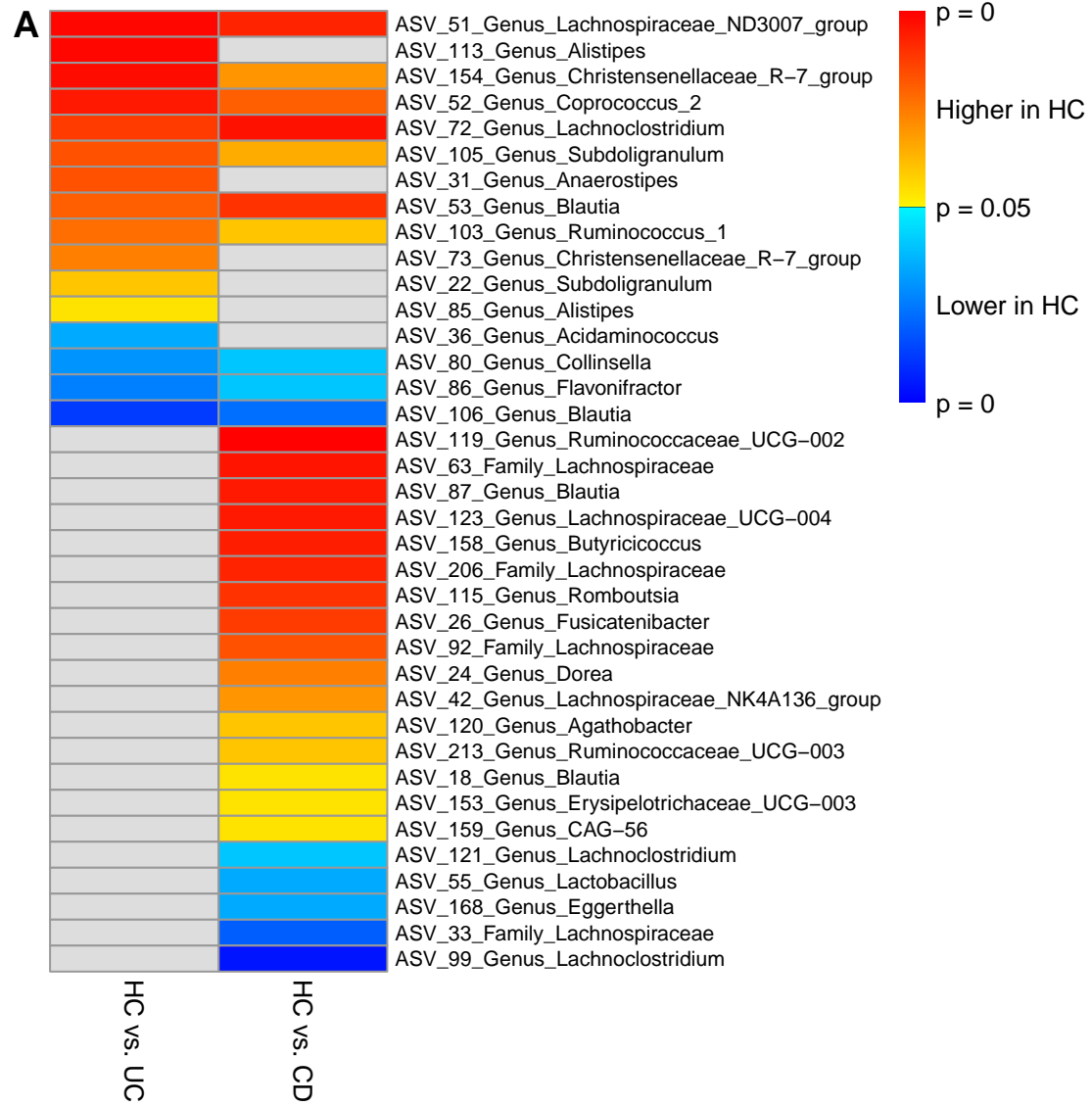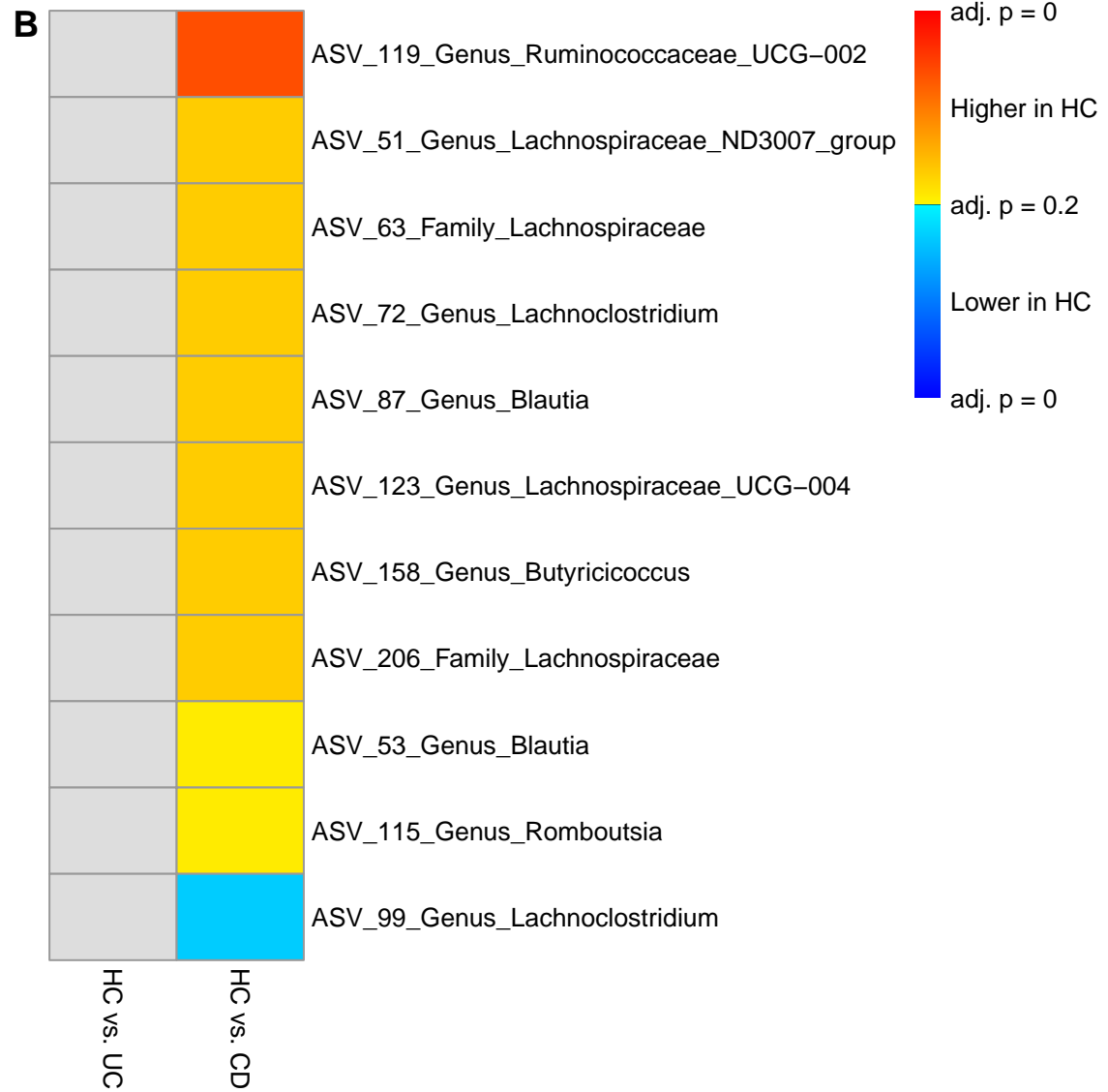

Supplement: Supplementary file 1 [file nutrients-14-01053-s001.zip › Figure S2.pdf]

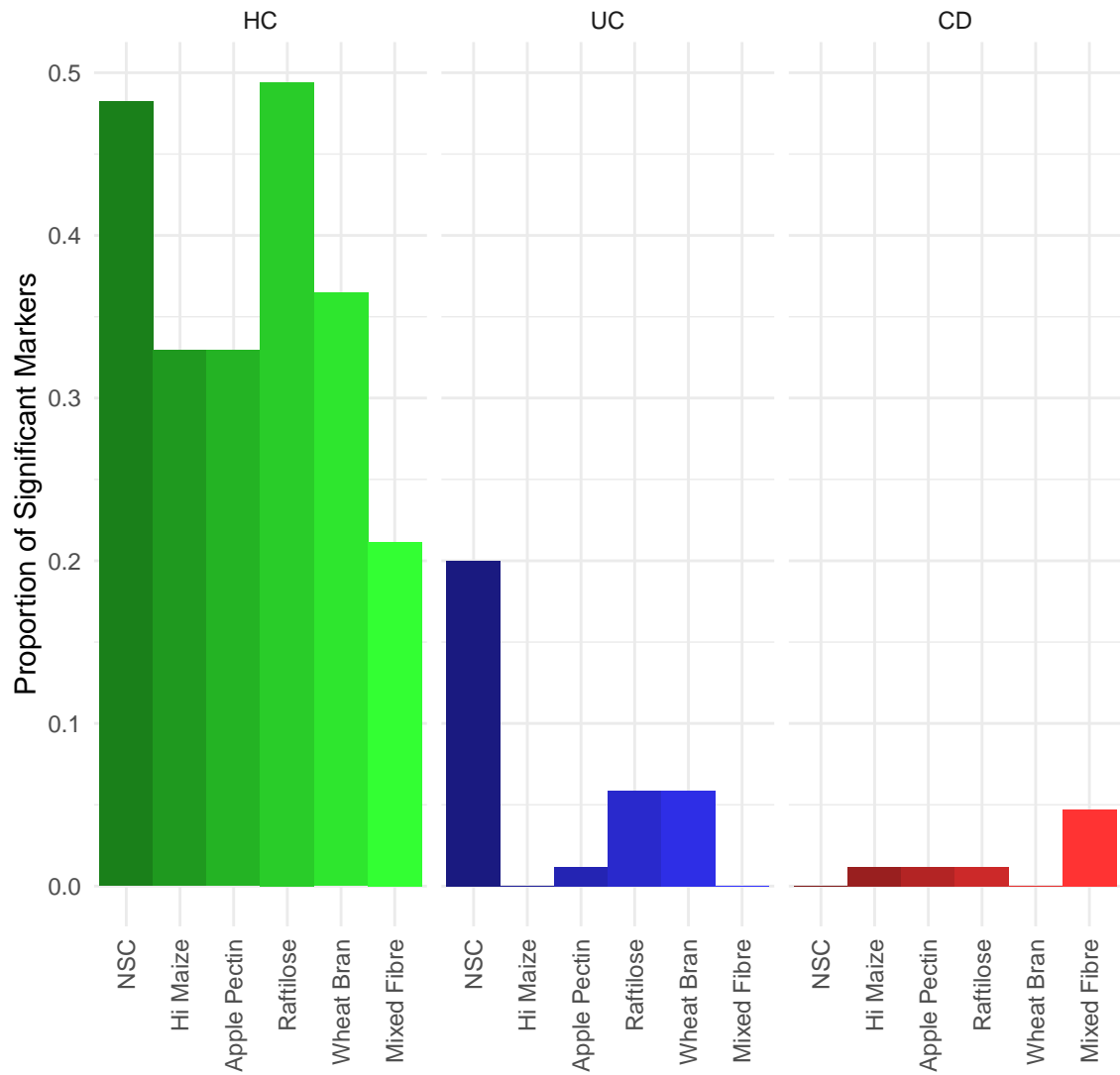

Supplement: Supplementary file 1 [file nutrients-14-01053-s001.zip › Figure S5.pdf]

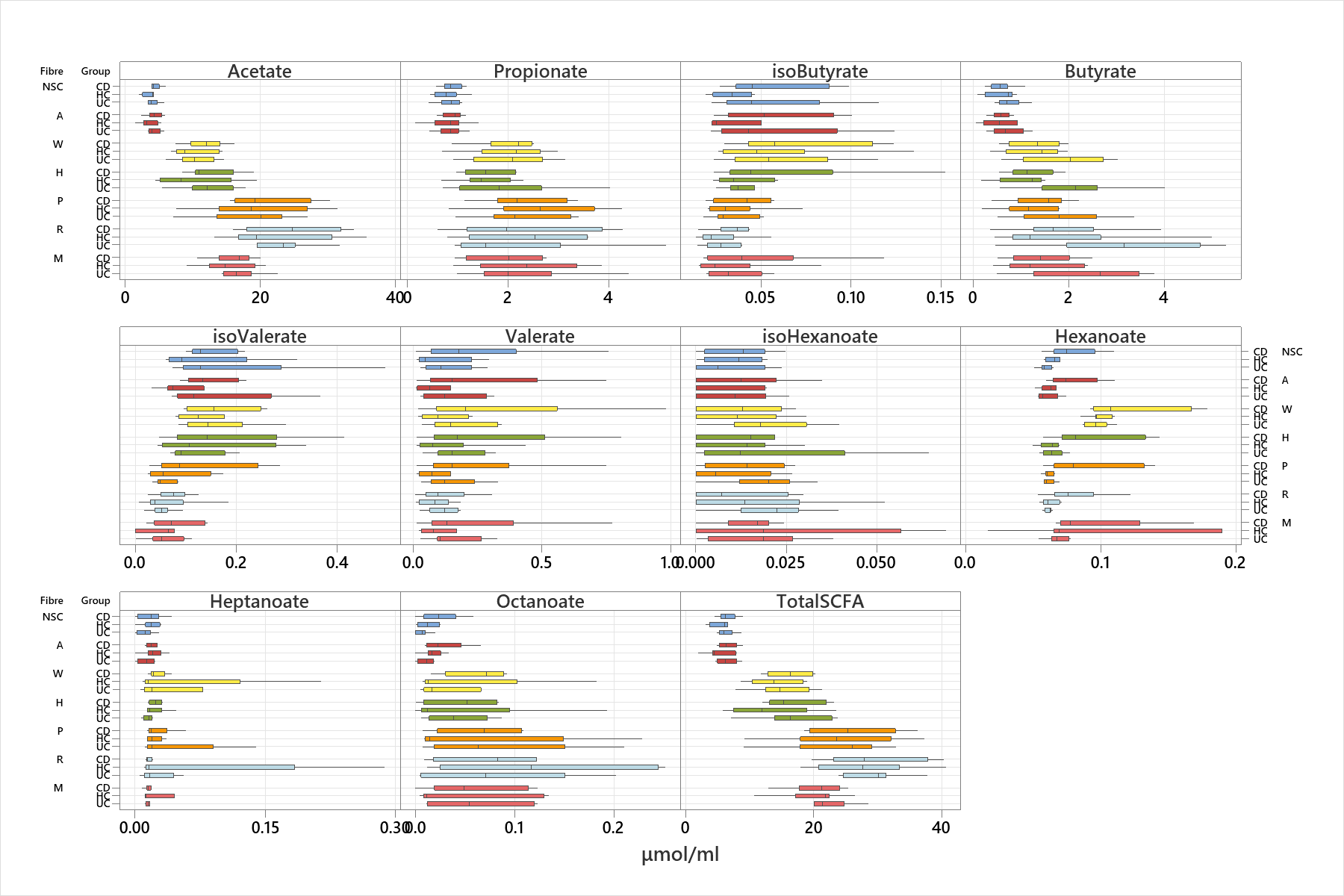

Supplement: Supplementary file 1 [file nutrients-14-01053-s001.zip › Figure S6.tif]
